# Mechanisms of antidiarrhoeal effects by diosmectite in human intestinal cells

**DOI:** 10.1186/s13099-017-0172-2

**Published:** 2017-04-24

**Authors:** Vittoria Buccigrossi, Carla Russo, Amedeo Guarino, Maiara Brusco de Freitas, Alfredo Guarino

**Affiliations:** 1 Department of Translational Medical Science, Section of Pediatrics, University of Naples Federico II, via S. Pansini 5, 80131 Naples, Italy; 2 Department of Nutrition, Federal University of Santa Catarina, Florianópolis, Santa Catarina Brazil

**Keywords:** Rotavirus infection, Diosmectite, Oxidative stress, Chloride secretion

## Abstract

**Background:**

Rotavirus (RV) induces diarrhoea through a sequence of enterotoxic and cytotoxic effects. The former are NSP4-dependent, induce calcium-dependent chloride secretion and involve oxidative stress. Diosmectite (DS) is a natural clay that has been recommended as an active therapy for diarrhoea, but the mechanism of its effect is not clear. Electrical parameters may be used to measure the direct enterotoxic and cytotoxic effects in polar epithelial intestinal cells. To investigate the effects of DS on RV-induced enterotoxic and cytotoxic damage. Caco-2 cells were used as a model of RV infection to evaluate chloride secretion, epithelial integrity, oxidative stress and viral infectivity in Ussing chambers.

**Results:**

Diosmectite reduced the expression of NSP4 and oxidative stress, resulting in a strong inhibition of chloride secretion. Preincubating RV with DS reduced the cytotoxic effect. Finally, the viral load was reduced by DS but not by control clay. This result suggests that DS specifically affects the early events of RV infection protecting the enterocyte, whereas it does not restore already-established cell damage.

**Conclusion:**

These findings indicate that DS exerts an anti-diarrhoeal effect by inhibiting viral replication and the expression of NSP4. Both ion secretion and cell damage induced by RV are strongly inhibited consequent to the antiviral effect, which explains its clinical efficacy.

## Background

Diosmectite (DS) is a natural clay that acts on the intestinal epithelium without being absorbed and exerts multiple effects in the intestine [1]. It interacts with the mucous layer and inhibits the mucolysis of intestinal epithelial cells in rabbit ileum infected with enteropathogenic *Escherichia coli* [2]. DS increases the resistance of the intestinal epithelium to toxic stimuli in humans [3]. It upregulates the colonic expression of MUC2, which is the main secretory mucin, thereby protecting the epithelium from the antigens produced during the inflammatory process [4]. In addition, DS restores the intestinal barrier function in an in vitro model of inflammation [5]. Previous studies have indicated that DS absorbs bacterial toxins, bacteria and viruses [6–8].

Diosmectite is proposed as an active treatment for acute gastroenteritis (AGE). The key treatment of AGE in children is the administration of oral rehydration solution (ORS) [9], but this neither shortens the duration of diarrhoea nor reduces the frequency of stool output. Therefore, active therapies are now recommended as an adjunct to ORS. The updated ESPGHAN/ESPID guidelines for managing children with gastroenteritis suggests the use of DS to reduce stool output [9] based on the results of randomized controlled clinical trials [10]. The latter have shown that DS reduces the stool volume in children with gastroenteritis, including those infected with RV [3, 11].

Rotavirus (RV) is the commonest aetiological agent of AGE in children and induces severe watery diarrhoea. Its severity is related to its mechanism of action, namely, a sequence of time-related mechanisms leading to secretory diarrhoea and intestinal epithelial damage [12]. In the early phase of infection, RV directly induces chloride and water secretion in the intestinal lumen through the enterotoxic effects of the non-structural viral protein NSP4. This increases the intracellular Ca^2+^ concentration and triggers electrogenic chloride secretion [12–14]. As recently reported, oxidative stress is a key mechanism in the enterotoxic effect induced by RV [14]. Following early ion secretion, RV infection results in severe damage to the structure of villi, with the disruption of epithelial integrity [15].

Clark et al. [8] demonstrated that aluminosilicate clays absorb a bovine rotavirus strain, but the infectivity rate was not inhibited in kidney epithelial cells. However, there are no data regarding the effects on RV infection.

The aim of this study was to evaluate the effects of smectite in a validated model of rotavirus diarrhoea in human-derived enterocytes in vitro [16]. Namely, we wanted to differentially investigate the effects of DS on intestinal epithelial damage and chloride secretion induced by RV infection, including the role of NSP4.

## Methods

### Human derived cell line

Caco-2 cells (ATCC Number: HTB-37) were used because they have the ability to differentiate into enterocytes of the upper villus forming monolayers. Cells were grown in high glucose (4.5 g/l) DMEM (Gibco, Life Technologies, UK) with 10% foetal calf serum (FBS) (Gibco, Life Technologies, UK), 1% non-essential amino acids, 50 mU/ml penicillin, and 50 mg/ml streptomycin. The Caco-2 cells were grown from 15 to 18 days after confluence on polycarbonate Transwell filters (pore size, 0.4 µm) (Costar Italia, Milan, Italy). MA104 cells (ATCC Number: CRL-2378) were used for viral titers and were grown in Medium 199 (Lonza, Belgium) with 5% FBS, 50 mU/ml penicillin, 50 mg/ml streptomycin, and 0.25 μg/ml amphotericin B.

### Adsorption assays

For adsorption assays, 100 mg/ml DS was incubated with the medium alone or in the presence of RV (MOI 25) for 1 h at 37 °C. Then, the viral suspensions were probed with fluorescein isothiocyanate (FITC) conjugated anti-RV antibody (Abcam, ab31435) and examined using a Nikon Eclipse 80i epifluorescence microscope (FITC filter). The images were analysed using the NIS Elements D imaging software. As a negative control, a mixture of titanium dioxide, maltodextrin and glucose monohydrate was used (TMG).

The same assay protocol was used to evaluate the absorptive effect of DS on NSP4. Briefly, 100 mg/ml DS was incubated with medium alone or in the presence of NSP4 at different doses (50, 100 and 200 ng/ml) for 1 h at 37 °C. The slides were probed with an anti-NSP4 antibody and then with a fluorescein isothiocyanate (FITC) conjugated anti-rabbit antibody. Then, the slides were examined using a Nikon Eclipse 80i epifluorescence microscope (FITC filter). The images were analysed using the NIS Elements D imaging software.

### RV positive cell staining

The Caco-2 cells were probed with FITC conjugated anti-RV antibody (Abcam, ab31435). Slides were mounted with Vectashield Mounting Medium with DAPI (Vector laboratories, Ltd, UK). The monolayers were examined using a Nikon Eclipse 80i epifluorescence microscope (FITC filter). The images were analysed using the NIS Elements D imaging software.

### Viral load assay

A fluorescence focus assay in the MA104 cell monolayers was used to determine the viral titre, expressed as focus-forming units per millilitre of virus (FFU/ml). Briefly, MA104 cells were grown in 8-chamber slides (Lab-Tek chamber Slide, Nunc Inc, USA) and then infected with supernatants from the Caco-2 infection experiments. After viral absorption, the cells were fixed in methanol and probed with fluorescein isothiocyanate (FITC) conjugated anti-RV antibody (Abcam, ab31435). Fluorescence foci were counted individually on a Nikon Eclipse 80i epifluorescence microscope (FITC filter). The viral titre was calculated from the average number of foci per well adjusted for well volume and expressed as FFU/ml. As a negative control, a mixture of titanium dioxide, maltodextrin and glucose monohydrate was used (TMG).

### Virus strain and Caco-2 cell infection protocol

The simian rotavirus strain SA11 (RV) (ATCC Number: VR-1565) was activated with 20 µg/ml trypsin for 1 h at 37 °C. The viral suspension was added to the apical side of the Caco-2 cell monolayer at a multiplicity of infection (MOI) of 25 to maximize the effect. After 1 h of incubation at 37 °C, the cells were rinsed three times and incubated in foetal calf serum–free medium for fixed times after infection. The time after infection was counted after the removal of excess viral particles. To test the effect of DS, RV was incubated with 100 mg/ml of DS for 1 h at 37 °C followed by centrifugation. The cells were then suspended and infected as previously described.

### Ion transport studies

Infection with RV was performed with multiplicities of infection (MOI) of 25 for 2 h; next, the cell monolayers were mounted in Ussing chambers (Physiological Instruments, San Diego, CA). The following electrical parameters were measured at different time points after infection: transepithelial potential difference (*PD*), short-circuit current (*I*sc), and tissue ionic conductance (*G*). *I*sc is expressed as microamperes per square centimetre (μA/cm^2^), *G* is expressed as millisiemens per square centimetre (mS/cm^2^), and *PD* is expressed as millivolts (mV). An increase in PD indicates chloride secretion and provides a precise evaluation of enterotoxic effects, whereas an increase in G quantitatively correlates with epithelial damage.

### Transepithelial electrical resistance measurements

The transepithelial electrical resistance (TEER) of cell monolayers grown on filters was measured using a Millicell-ERS resistance monitoring apparatus (Millipore). The net TEER (in Ohms/cm^2^) was calculated by subtracting the background from the actual value and multiplying the value obtained by the area of the filter (4.9 cm^2^).

### Western blot analysis

After RV infection, cells were scraped into PBS buffer and lysed in HEPES-KCl buffer (KCl, 60 mM; β-mercaptoethanol, 14 mM; EDTA, 2 mM; HEPES pH 7.9, 15 mM; sucrose, 0.3 M; aprotinin, 5 μg/ml; leupeptin, 10 μg/ml; pepstatin, 2 μg/ml; phenylmethylsulfonyl fluoride, 0.1 mM) containing 1% Tergitol (Nonidet P-40). Total extracts were centrifuged at 1500*g* for 20 min at 4 °C. The protein content was determined by the Bradford method (Bio-Rad Laboratories, Munich, Germany). The supernatant containing the solubilized proteins was boiled for 5 min in Laemmli buffer (62.5 mM Tris–HCl, pH 6.8, 2% SDS, 10% glycerol, 5% 2-mercaptoethanol, and 0.001% bromophenol blue). Cell protein (50 μg/lane) was added to SDS-PAGE and transferred to a nitrocellulose membrane (BioBlot-NC-Costar; Corning Incorporated, Canada). Blots were probed for 1 h with specific NSP4 antibody. Bound antibody was detected using an anti-rabbit immunoglobulin horseradish peroxidase-linked whole antibody and developed by chemiluminescence reaction (Amersham Pharmacia Biotech, U.K.). All incubations and washes were carried out at room temperature with gentle shaking.

### Reactive oxygen species (ROS) production

Reactive oxygen species production was measured using DCFH-DA spectrofluorometry. After stimulation, DCFH-DA (20 µM) was added for 30 min at 37 °C in the dark. Intracellular ROS production was measured in a fluorometer (SFM 25; Kontron Instruments, Japan). For DCF fluorescence imaging, Caco-2 cells were grown on the cover glass for 3 days, fixed and permeabilized with paraformaldehyde 4% and Triton 0.2% for 30 min at 4 °C. Cells were then incubated with DCF-HA 20 µM for 30 min at 37 °C in the dark. Fluorescence images from multiple fields were obtained using a Nikon Eclipse e 80i microscopy. The images were analysed using the NiS Elements D imaging software (Nikon Instruments, Inc., NY, USA).

### Glutathione assay

The intracellular GSH/GSSG ratio was determined by a fluorimetric assay kit (Biovision, Milpitas, CA). The GSH content was normalized for protein content and expressed as % of control.

### Statistical analysis

We used the GraphPad Prism Software (San Diego, CA) to evaluate the two-tailed unpaired student *t* test and a 2-tailed paired student *t* test to evaluate statistical significance. An alpha value of 0.05 was set for statistical significance; p values for each analysis are indicated in the figure legends. All experiments were repeated at least three times, and error bars indicate the standard deviation.

## Results

### Absorption of RV virions by DS

**Fig. 1:**
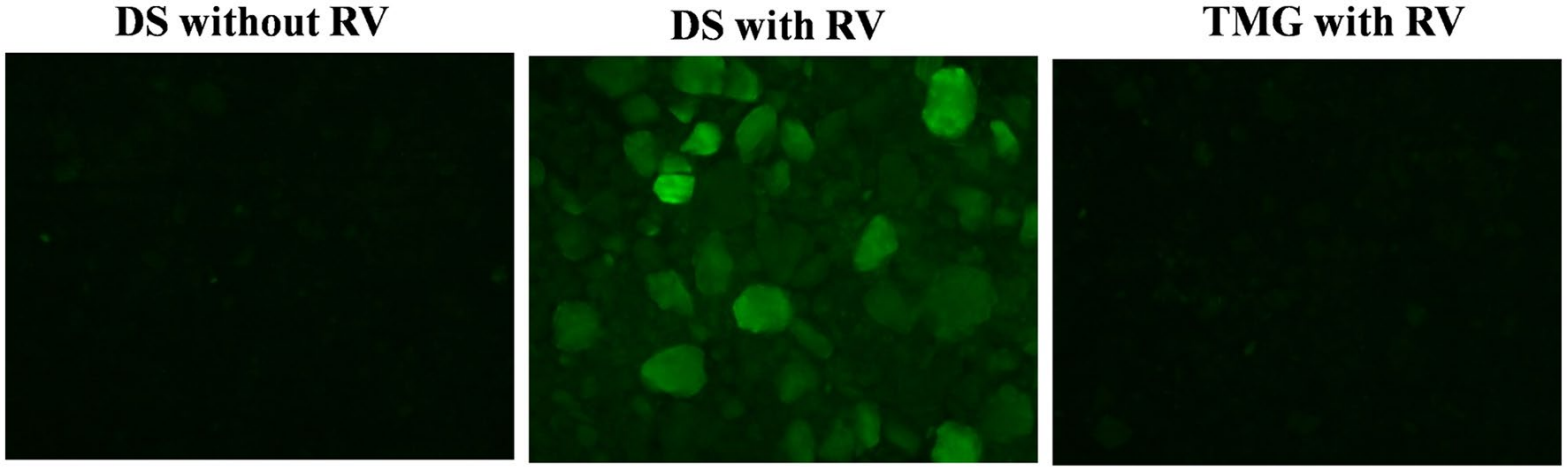
Binding of RV to DS. DS was incubated with RV, and the suspension was then probed with fluorescein isothiocyanate (FITC) conjugated anti-RV antibody as described in the “Methods”. DS alone or TMG incubated with RV was used as negative controls. Images are representative of three separate experiments

First, we tested the ability of DS to bind RV particles. After 1 h of incubation, we found that DS bound RV virions as judged by specific RV fluorescence, whereas the control clay TMG incubated with RV did not show any presence of virus (Fig. 1). The experiments were repeated by incubating pure NSP4 with DS, and no binding was observed between NSP4 and DS (data not shown).

### Effects of DS on RV infectivity

**Fig. 2:**
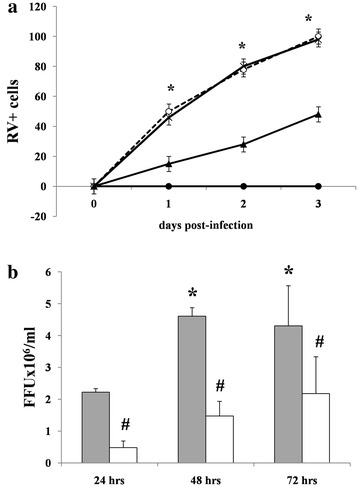
Effect of DS on RV infectivity. Caco-2 cells were infected with RV with or without DS for 3 days. **a** Quantitative analysis of RV-infected Caco-2 cells. Caco-2 cell monolayers were infected with RV preincubated in the presence (*filled triangle*) or absence (*open circle*) of DS as described in the “Methods”, and the RV-infected cells (RV+) were counted. TMG (*times*) was used as the negative control together with uninfected cells (*filled circle*) [*p < 0.05 vs DS-treated RV]. **b** Viral titer of the culture medium of RV-infected Caco-2 cells alone (*grey*) or preincubated with DS (*white*) [*p < 0.05 vs RV at 24 h;^#^p < 0.05 vs RV at the same time]

To investigate the effects of DS on RV infection further, we evaluated the number of RV-infected cells. After 3 days of RV infection in Caco-2 cells, the relative number of RV-positive cells was substantially reduced when RV was pretreated with DS (Fig. 2a). In addition, DS significantly reduced the number of virions available for infection compared with untreated RV (Fig. 2b). The number of RV particles was always lower compared with DS-untreated parallel cells infected with RV.

### Effects of DS on RV-induced chloride secretion

**Fig. 3:**
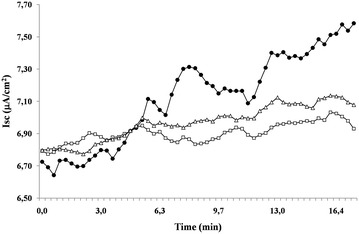
Effects of DS on the enterotoxic effect induced by RV. Caco-2 cell monolayers were infected with RV preincubated in the presence (*open triangle*) or absence (*filled circle*) of DS for 2 h as described in the “Methods”, and then the short-circuit current (*I*sc) was evaluated in Ussing chambers together with uninfected cells (*open square*). This is a single representative experiment

**Fig. 4:**
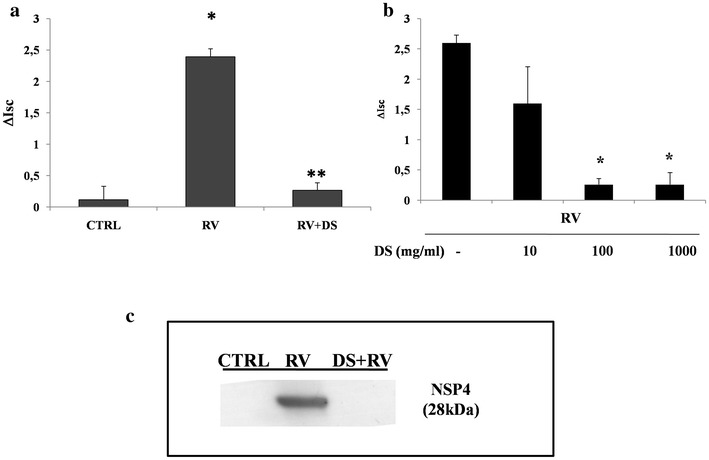
DS prevents the enterotoxic effect induced by RV. **a** Maximal effect by RV on *I*sc. Three independent experiments were pooled to analyse the statistical significance [*p < 0.05 vs control; **p < 0.05 vs RV]. **b** DS totally inhibited the enterotoxic effect of RV on the *Isc* in a dose-dependent manner [*p < 0.05 vs RV alone]. **c** NSP4 expression was substantially inhibited by pretreatment with DS

As previously reported in the basic model of Caco-2 cells, RV infection induced chloride secretion, which was NSP4-dependent and involved oxidative stress [12, 14]. RV was treated with 100 mg/ml DS for 1 h before cell infection; then, the RV was incubated with Caco-2 cells in the infection phase for 2 h, and the *I*sc was measured. The *I*sc did not increase in DS-treated RV (Figs. 3, 4a), which suggests that DS effectively counteracts RV-induced chloride secretion. The effect was dose-dependent, and DS was effective in a concentration range of 100–1000 mg/ml (Fig. 4b).

Since RV induces chloride secretion through NSP4 [12, 14], we evaluated whether DS affected NSP4 expression. Figure 4c shows a representative experiment in which NSP4 expression was virtually abolished in the presence of DS.

### Effects of DS on RV-induced oxidative stress

**Fig. 5:**
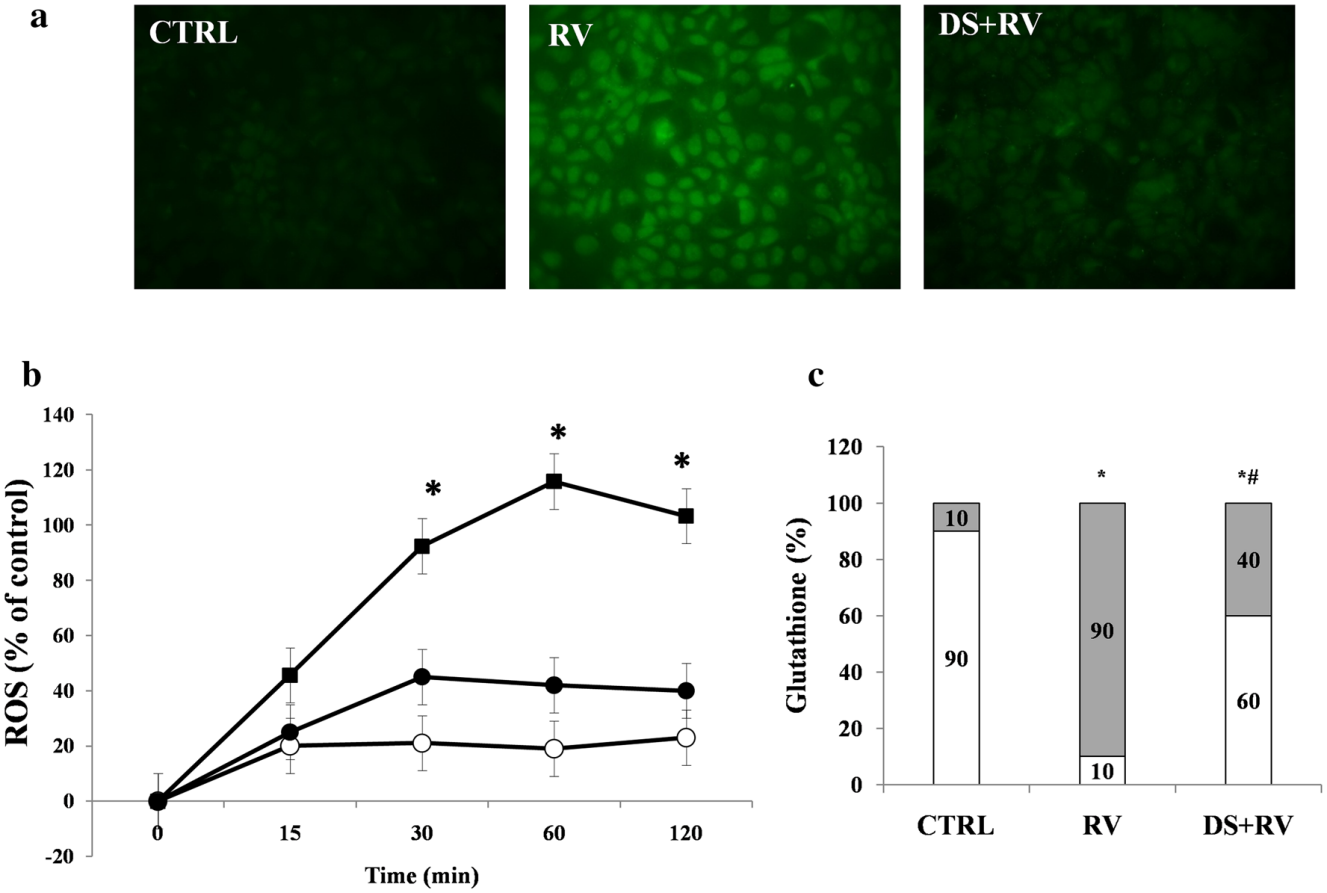
Effect of DS on oxidative stress induced by RV. **a** Caco2 cells were infected with RV following preincubation in the absence or presence of DS, and the fluorescence of the ROS probe was evaluated 1 h following the infection. Magnification: 400×. **b** Time-course of ROS production. Caco-2 cell monolayers were infected with RV preincubated in the presence (*filled circle*) or absence (*filled square*) of DS as described in the “Methods”, and the ROS intracellular levels were evaluated together with uninfected cells (*open circle*) [*p < 0.05 vs the control at the same time]. **c** Caco-2 cell monolayers were infected with RV, glutathione was evaluated 1 h following the infection, and the levels of GSH (*grey*) and GSSG (*white*) were measured. DS was present during the activation phase of the virus, as described in the “Methods”. *p < 0.05 vs control;^#^p < 0.05 vs RV

Because RV-induced enterotoxic damage is oxidative stress-dependent [14], the redox state was evaluated in Caco-2 cells infected with RV following preincubation in the absence or presence of DS. RV induced ROS production as indicated by an increase in the green signal of DCF-DA on fluorescence microscopy. DS effectively prevented oxidative stress (Fig. 5a).

The time-course of the inhibitory effect of DS on ROS production was evaluated to determine whether it was maintained over the course of the experiment, and significant inhibition was indeed observed over the entire course of infection (Fig. 5b). To evaluate the role of antioxidant defences, we determined the levels of reduced and oxidized glutathione (GSH/GSSG ratio) in RV-infected Caco-2 cells following preincubation in the absence or presence of DS. The GSH/GSSG ratio was reduced when the virus was exposed to DS compared to controls (Fig. 5c).

### Effects of DS on RV-induced cytotoxic damage

**Fig. 6:**
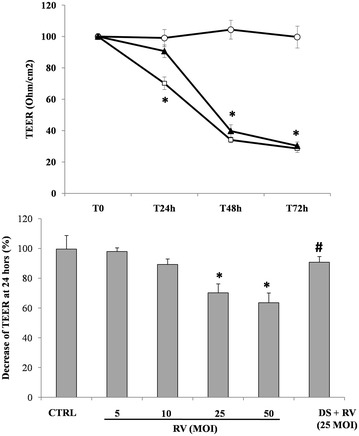
Effect of DS on cytotoxic damage induced by RV. **a** Caco-2 cells monolayers were infected with RV following preincubation in the presence or absence of DS for 48 h. Caco-2 cell monolayers were infected with RV following preincubation in the presence (*filled triangle*) or absence of DS (*open square*) and the transepithelial electrical resistance (TEER) was evaluated as described in the “Methods” together with uninfected cells (*open circle*); *p < 0.05 vs the control at the same time. **b** Effect of DS on RV-induced cytotoxic damage compared with an increasing viral load. *p < 0.05 vs control; ^#^p < 0.05 vs RV 25 and 50 MOI

We evaluated TEER, a measure of epithelial integrity, in time-course experiments (Fig. 6a). RV infection produced a reduction in TEER, whose intensity was related to the viral load (MOI) (Fig. 6b). The decrease in TEER was observed 24 h post-infection and reached a plateau at 48–72 h post-infection. In the presence of DS, a delay in the decrease of TEER was observed. However, in prolonged experiments, the pattern of TEER decrease obtained with a high load of RV in the presence of DS became similar to that observed with a low viral load in the absence of DS (Fig. 6b).

## Discussion

RV-induced diarrhoea is the result of multiple combined mechanisms. RV targets mature enterocytes, altering the villous structure and inducing disaccharidase loss and carbohydrate malabsorption. It damages tight junction structure and impairs the barrier function of intestinal epithelial cells, allowing RV to access the basolateral side of the enterocyte [17].

Watery diarrhoea results from increased chloride secretion mainly induced by the viral enterotoxin NSP4 [18], which stimulates cAMP-dependent Cl^−^ secretion by increasing the intracellular Ca^2+^ concentration [18] and inhibiting Na^+^ absorption [13], ultimately causing the activation of the TMEM16A transporter protein [19]. However, the chloride conductance of CFTR is unaffected by NSP4 [19].

Diosmectite binds several bacterial and viral toxins [6, 7], and this has been postulated to contribute to its antidiarrhoeal effect. Clark et al. [8] observed that different clays absorb a bovine RV strain, but the infectivity was actually increased. The authors speculate that this unexpected phenomenon is the result of the more efficient presentation of virus by clays, supporting virus carriage into the cell by the clay, but no studies have been conducted to support this hypothesis. The increased infectivity was observed in kidney epithelial cells, but no information is available regarding the intestinal epithelium.

In our experimental approach, we separately investigated the effects induced by DS on the enterotoxic and cytotoxic pathways of RV-induced diarrhoea in a human model. Our results indicate that DS effectively reduces the intensity of RV infection of intestinal epithelial cells and inhibits RV-induced chloride secretion in Caco-2 cell monolayers. This effect is the result of an interaction between DS and RV, whereby the adsorption of RV particles by DS effectively reduces the infection load on epithelial cells. The subsequent reduction of chloride secretion observed in our in vitro model provides a plausible explanation for the reduction of stool output in children with acute gastroenteritis treated with DS [10].

We have previously demonstrated that NSP4 exerts severe direct enterotoxic effects by modifying the cellular redox state [14], and the findings of the present study indicate that DS is effective in reducing NSP4 expression and can thus protect epithelial cells from oxidative stress.

However, the DS effect is due to the reduction of the viral load rather than the binding to specifically inhibit NSP4 production since DS was not able to bind NSP4 as such.

Rateau et al. [20] described the anti-diarrhoeal effects of DS related to increased chloride and magnesium absorption in the rabbit ileal mucosa infected with enterotoxigenic *E. coli*. These effects were postulated to be due to the inhibition of mucolysis or to the attenuation of damage to the luminal surface of the intestinal mucosa. In addition, DS reduced interleukin-1β (IL-1β) secretion and decreased neutrophil infiltration and monocyte activation, which both contributed to a reduction of the antigenic load in hapten-induced colitis in the rat [4]. Since IL-1β induces chloride secretion [21], it is possible that this anti-inflammatory effect contributes to the antidiarrhoeal mechanism of DS. However, such a mechanism cannot explain the effects observed with RV diarrhoea, which is not associated with intestinal inflammation.

The cytotoxic effect of RV on epithelial cells occurs later than the acute effect on chloride secretion, and it was observed only 1–2 days after the initial infection in our experimental model [12]. The efficacy of DS in protecting the integrity of the intestinal epithelium demonstrated in several previous studies was thought to be the result of the clay interacting with the intestinal mucosa. In this respect, it has been reported that DS acts as a physical barrier to toxic stimuli such as proinflammatory mediators [5], facilitates the secretion of MUC2 into the colon and increases the thickness of mucus [4].

In the present study, we show that DS binds RV, thereby reducing the viral load and virion release from infected cells. Although the remaining viral load was still sufficient to cause cell damage and spread infection in our model, the structural (cytotoxic) and functional (enterotoxic) consequences of RV infection were both strongly reduced. In particular, the number of Caco-2 cells infected with RV decreased in the presence of DS. In addition, the number of virions released in the culture medium was lower in cells infected with DS-treated RV rather than RV alone. This protective effect was maintained over three days following the infection. We therefore suggest that the anti-diarrhoeal effects of DS against RV are associated with a specific capacity of this clay to bind the virus, thus blocking both viral replication and the pathogenic effects of NSP4.

The results reported in this paper provide a novel explanation for the antidiarrhoeal effects observed with DS in children. RV is the main agent of infantile diarrhoea and is by far the most dangerous. However, DS is clinically effective against diarrhoea irrespective of the aetiology [11], and there is a need to understand whether the mechanisms described in this paper, which essentially consists of trapping viral particles and blocking downstream pathogenic events, may explain a more general effect against enteric pathogens. In addition to classic enterotoxic pathogens, several agents induce diarrhoea in all or in part by enterotoxic moieties, including *Cryptosporidium* and HIV virus [22]. If these results are also confirmed with these agents, an entirely new understanding of the antidiarrhoeal effects of DS may open new options for treating diarrhoea. This may also include inflammatory diarrhoea, as the latter is induced by endogenous secretagogues, particularly inflammatory cytokines or others inflammatory gut disease [23]. Clinical evidence of an effect by DS in inflammatory diarrhoea has been reported [11]. Similar considerations apply to endocrine diarrhoea and diarrhoea due to radiotherapy.

Diosmectite possesses a powerful anti-diarrhoeal effect against RV and reduces chloride secretion, which is responsible for the observed decrease in stool output. These results support the inclusion of DS as an effective option for the active treatment of diarrhoea (as suggested in authoritative guidelines) and open new perspectives for using DS in other forms of diarrhoea.
